# Development of Multiplex PCR Coupled DNA Chip Technology for Assessment of Endogenous and Exogenous Allergens in GM Soybean

**DOI:** 10.3390/bios11120481

**Published:** 2021-11-26

**Authors:** Tamara Kutateladze, Kakha Bitskinashvili, Nelly Sapojnikova, Tamar Kartvelishvili, Nino Asatiani, Boris Vishnepolsky, Nelly Datukishvili

**Affiliations:** 1 Ivane Beritashvili Center of Experimental Biomedicine, 14 Gotua str., Tbilisi 0160, Georgia; kutateladzet@yahoo.com (T.K.); kakha.bitskinashvili.1@iliauni.edu.ge (K.B.); b.vishnepolsky@gmail.com (B.V.); 2 School of Natural Sciences and Medicine, Ilia State University, 3/5 Kakutsa Cholokashvili Ave, Tbilisi 0162, Georgia; 3 Andronikashvili Institute of Physics, I. Javakhishvili Tbilisi State University, 6 Tamarashvili Str., Tbilisi 0162, Georgia; nelly.sapojnikova@tsu.ge (N.S.); tamar_kart@yahoo.com (T.K.); nina_asatiani@yahoo.com (N.A.)

**Keywords:** food allergen, genetically modified soybean, allergen detection, multiplex PCR, DNA microarray

## Abstract

Allergenicity assessment of transgenic plants and foods is important for food safety, labeling regulations, and health protection. The aim of this study was to develop an effective multi-allergen diagnostic approach for transgenic soybean assessment. For this purpose, multiplex polymerase chain reaction (PCR) coupled with DNA chip technology was employed. The study was focused on the herbicide-resistant Roundup Ready soya (RRS) using a set of certified reference materials consisting of 0, 0.1%, 0.5%, and 10% RRS. Technically, the procedure included design of PCR primers and probes; genomic DNA extraction; development of uniplex and multiplex PCR systems; DNA analysis by agarose gel electrophoresis; microarray development, hybridization, and scanning. The use of the asymmetric multiplex PCR method is shown to be very efficient for DNA hybridization with biochip probes. We demonstrate that newly developed fourplex PCR methods coupled with DNA-biochips enable simultaneous identification of three major endogenous allergens, namely, Gly m Bd 28K, Gly m Bd 30K, and lectin, as well as exogenous 5-enolppyruvyl shikimate-phosphate synthase (epsps) expressed in herbicide-resistant roundup ready GMOs. The approach developed in this study can be used for accurate, cheap, and fast testing of food allergens.

## 1. Introduction

Soybean (*Glycine max*) is an edible legume distributed worldwide. It is widely used as both a raw material and an ingredient in food production. In recent years, soya consumption has increased because it is utilized as an emulsifier, texturizer, and protein filler, as well as meat analogs and milk substitutes. It substantially improves the nutritional and functional properties of food products [1]. However, soybean is considered one of the eight most prevalent food allergens. In recent decades, according to the available data of epidemiology, pathogenesis, diagnosis, and treatment, food allergies have increased. They can cause various symptoms ranging from mild to severe including anaphylaxis and, thus, food allergy is a potentially life-threatening illness. Due to the absence of a cure, food allergies are recognized as a significant rising problem for public health. Thus, sensitive individuals should avoid consumption of allergenic foods to prevent allergic reactions [2]. In order to support food safety and public health, the international and local regulations necessitate labeling of food allergens. Provision of accurate information about the presence of food allergens through label declarations is essential for the implementation of legislation and assistance of allergic consumers. The list of the priority allergenic foods varies among countries, although labeling of soybean is mandatory by the international FAO/WHO codex allimentarius general standard, as well as EU and national regulations [3,4].

The allergenicity of genetically modified foods is of particular interest. The genetically modified organisms (GMOs) produced by modern biotechnology exist as plants, seeds, grains, foods, feeds, and other products. In recent decades the planting area of transgenic plants has been continuously increased [5]. The most prevalent transgenic crops are herbicide-tolerant and insect-resistant soybean, maize, cotton, and rapeseed. The genetically modified (GM) plants possess beneficial traits however they have potential health risks including allergens and toxins. Therefore, an assessment of allergens in GM plants and foods is requested by the international food legislation such as codex alimentarius. The GMO allergenicity is evaluated according to the European Food Safety Authority (EFSA) Guidance for the risk assessment of genetically modified plants and derived food and feed [6,7,8]. To implement regulations, reliable and effective methods for GMO allergen detection are needed.

The allergen detection is based on the analysis of two main targets: allergy-inducing proteins and/or their encoding DNA. At present, different approaches have been developed for allergenic soybean detection including ELISA, PCR, and MS [9,10,11,12]. The studies of allergen-specific immunoglobulin IgE revealed 15 allergenic proteins of soybean. Four of these were identified as major allergens, such as Gly m 5 (beta-conglycinin), Gly m 6 (glycinin), Gly m Bd 28K (26kDa glycoprotein), and Gly m Bd 30K (peptidase C1) [13,14]. The individual allergenic proteins of soybean have been mainly investigated by immunological approaches. The suitable ELISA methods for their detection have been developed and described [14,15,16,17,18]. However, ELISA and MS techniques are both time-consuming and expensive. DNA-based methods have high specificity and reliability for allergen determination. PCR is preferably used to control allergenic ingredients in different foodstuffs since DNA is a more stable molecule than protein during food processing. The main advantage of the PCR technique is its specificity, reliability, and rapidity allowing the detection of trace amounts of allergens in processed products. The end-point and real-time PCR methods have been reported for the detection of soybean allergens, namely Gly m Bd 28K [19], Gly m Bd 30K [20], lectin [21]. The comparison of ELISA and PCR methods using soya food products showed that the sandwich ELISA had the lowest detection limit of 0.05 ppm, but only identified soy in five out of the eight products. The competitive ELISA had a higher detection limit of 21 ppm but seemed more successful in detecting processed soy. However, PCR detected soy in all eight products [22].

GM soybean is the most widely distributed transgenic crop, in addition, it is the only of the current nine commercially available GM crops among the ‘big eight’ allergenic foods [23]. The introduction of new genes from other organisms into the plant genome resulted in the expression of new, potentially allergenic proteins in transgenic plants. Thus, the determination of GMO allergens implies an assessment of both the species-specific endogenous and newly introduced exogenous allergens. The assessment of both the potential growth of endogenous allergens and the detection of the newly expressed protein in GMO is requested by the Codex Alimentarius [24,25]. The comparison of the levels of the eight soybean allergens did not show important differences in the GM and non-GM soybeans using ELISA [26].

The multiplex approaches including multiplex PCR, MS as well as microarrays and biosensors allow simultaneous detection of several allergens in one test. Correspondingly, they enable to reduce the costs and the duration of the experiment. Moreover, the cross-contamination can be decreased using microarrays and biosensors [27,28,29,30]. To date, only a few studies have described multiplex technologies for food allergens including soybean. The digital versatile disk was used to develop DNA microarray joined with the multiplex PCR technique for the parallel determination of hazelnut, peanut, and soybean in foods [27]. Three important allergenic crops such as sesame, peanut, and soybean were detected by triplex Q-PCR [31]. Furthermore, ten soybean allergens were assessed by tandem mass spectrometry [32].

Despite the availability of different techniques, the selection of an appropriate method for allergen detection can be challenging, due to the complexity and processing of food material. There are a lot of problems and unresolved issues regarding sampling strategies; extraction methods; reference materials; food matrix, DNA and protein degradation, multiplexing, and the economic impact of testing. The monitoring system requires efficient methods for detecting many allergens in a time-saving and cost-effective manner.

In the present study, an approach of multiplex asymmetric PCR coupled with a biochip was developed for the first time as a rapid and cheap tool for the detection of endogenous and exogenous allergens in GM soybean. The presented approach allows simultaneous identification of three major endogenous allergens, such as Gly m Bd 28K (glycoprotein), Gly m Bd 30K (peptidase C1), and lectin (Le1) as well as exogenous 5-enolppyruvyl shikimate-phosphate synthase (epsps) expressed in herbicide-resistant roundup ready GMOs. The methodology has been validated using certified reference materials (CRMs) of Roundup Ready soya. The methods obtained may be readily applied for accurate, low-cost, and fast testing of food allergens.

## 2. Materials and Methods

### 2.1. Plant Material

The set of GM Soya bean CRMs containing 0, 0.1%, 0.5%, and 10% Roundup Ready soya (ERM-BF-410) were purchased commercially (Sigma-Aldrich, Merck, Darmstadt, Germany). The locally produced seeds of unmodified soybean (*Glycine max*) were obtained from a local market in Tbilisi (Georgia). The soybean seeds were ground by an electric grinder (Siemens, Munich, Germany) to obtain flour. The GM soya powders were used directly for DNA extraction.

### 2.2. DNA Extraction

DNeasy plant mini kit (Qiagen, Hilden, Germany) was chosen as a suitable procedure for DNA extraction based on the results of our previous study [33]. Genomic DNAs were isolated and purified from 100 mg flour. The purity and concentration of the extracted DNAs were estimated by spectrophotometer DeNovix DS-11 (DeNovix Inc. Wilmington, NC, USA). The quantity and integrity of DNA were evaluated using electrophoresis (VWR International, Radnor, PA, USA) on 1% agarose gel (SeaKem LE agarose; Cambrex, East Rutherford, NJ, USA) containing 1 μg/mL of ethidium bromide (Sigma-Aldrich, St. Louis, MI, USA). The agarose gels were visualized under ultraviolet (UV) light and a digital image was obtained using a gel documentation system PhotoDoc- It imaging system (UVP, Upland, CA, USA).

### 2.3. Bioinformatic Analysis, Design of Oligonucleotide Primers and Probes

The available literature, protein (https://www.uniprot.org, accessed on 19 January 2021), and allergen (http://www.allergome.org, https://fermi.utmb.edu/SDAP/, accessed on 19 January 2021) databases, were screened in order to identify important allergens for soybean as well as roundup ready soya. The corresponding DNA sequences were extracted from GenBank (https://www.ncbi.nlm.nih.gov/nucleotide, accessed on 25 January 2021). We selected three important soybean food allergens and identified their gene sequences, such as Glycine max Gly m Bd 28K-glycoprotein (GenBank acc.no. EU493457), Glycine max Gly m Bd 30K-peptidase C1 (GenBank acc. no. FJ616287.1), and lectin (GenBank acc. no. K00821.1). In addition, the gene producing potential transgenic allergenic protein 5-enolppyruvyl shikimate-phosphate synthase (*epsps*) (GenBank acc. no. AB209952.1) was monitored. The different primer pairs were designed using available web-services and standalone tools. Firstly, the PCR primers and microarray oligos were chosen by Primer-BLAST [34] and PrimerQuest tool (https://eu.idtdna.com/PrimerQuest, accessed on 17 February 2021). In addition, the sequence alignment tool Align_MTX [35] was used for the final design whereby, the possible formation of dimers and secondary structures was evaluated by FastPCR [36]. The fitness of the primer pairs for the multiplex PCR system was checked using MultiPLX [37] and FastPCR. The PCR primers and microarray probes used in this study are shown in Table 1.

The primer pair Lect101f/Lect101r targeting the soybean lectin gene was taken from our previous publications [38]. The oligonucleotide primers for PCR were synthesized and purified by Integrated DNA Technologies (Coralville, IA, USA). While the labeled primers and probes for chip development and analysis were prepared and provided by Eurofins Genomics (Ebersberg, Germany).

### 2.4. PCR Analysis

The PCR amplifications were carried out on the thermal cycler Techne TC-412 (Techne, Minneapolis, MN, USA). The several parameters, namely, MgCl_2_ concentration (1.0–6 mM), primer concentration (0.1–1.5 μM), annealing temperature (52–68 °C), elongation time (20–90 s), and cycle number (30–60) were verified to optimize PCR conditions.Each 25 μL uniplex PCR reaction included 1× Taq buffer, 1.5 mM MgCl_2_, 0.2 mM dNTP, 1.25 U Taq DNA polymerase (New England BioLabs, Ipswich, MA, USA), 50 ng of genomic DNA, and 0.5 μM of each primer. The fourplex PCRs were performed in 25 μL, consisting of multiplex PCR master mix with 1.25 U HotStarTaq DNA polymerase (Qiagen, Hilden, Germany), 3 mM MgCl_2_, 50 ng of genomic DNA, and the primer concentrations as follows: 0.3 μM for each of the primers targeting to the endogenous genes and 0.8 μM for each of the primers: epsps172f, epsps172r, epsps114f, and epsps114r. Sterile water was used as a negative control in all PCR analyses.

The uniplex PCRs with primer pairs 28K86f/28K86r, 28K98f/28K98r, 30K118f/30K118r, 30K129f/30K129r, and 30K122f/30K122r revealed the same optimal cycling profile, such as preincubation at 95 °C for 3 min, 40 cycles consisting of DNA denaturation at 95 °C for 30 s, primer annealing at 60 °C for 30 s, elongation at 72 °C for 35 s; final extension step at 72 °C for 5 min. The PCR cycling conditions for primer pairs Lect101f/Lect101r and lect135f/lect135r were as follows: denaturing at 95 °C for 5 min, 50 cycles of 30 s at 95 °C, 30 s at 63 °C, 30 s at 72 °C; final extension at 72 °C for 3 min. The same amplification profile was optimized for primer pairs epsps114f/epsps114r and epsps172f/epsps172r, such as denaturing at 95 °C for 5 min, 40 cycles of 30 s at 95 °C, 35 s at 60 °C, 45 s at 72 °C; final extension at 72 °C for 5 min.

All multiplex PCRs were performed by the same optimized amplification profile as follows: preincubation for 15 min at 95 °C, 40 cycles of 30 s at 95 °C, 45 s at 60 °C, 60 s at 72 °C; final extension step for 7 min at 72 °C. The uniplex and multiplex PCR products were detected by electrophoresis on 2.0% or 3.5% agarose gels respectively; afterwards they were visualized under ultraviolet (UV) light and photographed using a gel documentation system PhotoDoc-It imaging system.

### 2.5. Asymmetric Multiplex PCR and Labelling

The asymmetric fourplex PCRs were carried out by the same above-mentioned optimized protocol for multiplex PCR (2.4 PCR analysis) except for the primer labeling and concentration. In particular, the Reverse primers (Lect101; Lect135; epsps114; 28K86; 28K98; 30K118; 30K122 and 30K129) and forward primer (epsps172) were Cy3 labeled at the 5′-end (Table 1) for the visualization of PCR products on a biochip. In addition, the ratio of labeled primer concentration to unlabeled primer concentration was optimized to get a sustainable hybridization signal on a biochip being 6:1 for each pair. In particular, the concentration of all labeled R primers (Lect135, Lect101, 28K98, 28K86, 30K122, 30K129, and 30K118) were 0.6 μM and all corresponding F primers were 0.1 μM per reaction, however, the concentration of the labeled F primer Epsps172 was 1.6 μM and R primer was 0.26 μM per reaction while the concentration of the labeled R primer Epsps114 was 1 μM and F primer was 0.16 μM per reaction. Therefore, the single-stranded (ss) Cy3-labelled PCR products prevailed in the asymmetric PCR. The sequences of the probes are complementary to the reverse or forward chain of Cy3-labelled DNA, providing for the visualization after hybridization.

### 2.6. Biochip Preparation and Analysis

The biochip technology, the conditions of DNA preparation, and hybridization on a biochip were the same as described previously [39]. The chips were prepared using oligonucleotide probes described in Table 1. Four types of DNA chips were made including different combinations of oligonucleotide probes while each chip contained oligos corresponding to four allergens, namely, Gly m Bd 28K, Gly m Bd 30K, lectin, and epsps. The asymmetric multiplex PCR products were used as the amplified and labeled DNA in the hybridization with the biochips including the suitable oligos.

The samples were tested at least in duplicate in each PCR and biochip analysis in three independent experiments. The comparison of the outcomes revealed the reproducibility of the results.

## 3. Results

### 3.1. Identification of Effective PCR Primers for Detection of RRS Allergens

**Figure 1:**
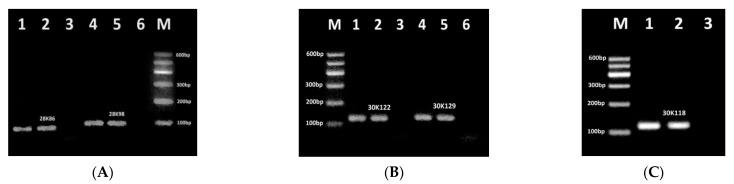
PCR detection of soybean allergens: Gly m Bd 28K using primer pairs 28K86f/28K86r (**A**, lanes 1–3) and 28K98f/28K98r (**A**, lanes 4–6), Gly m Bd 30K using primer pairs 30K122f/30K122r (**B**, lanes 1–3), 30K129f/30K129r (**B**, lanes 4–6), 30K118f/30K118r (**C**, lanes, 1–3). Samples: lanes 1–2, 4–5. soybean seeds flour, lanes 3, 6. water (negative control). M. Molecular weight marker (Qiagen GelPilot 100 bp ladder).

**Figure 2:**
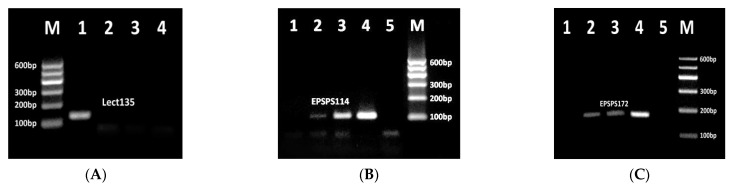
PCR detection of soybean allergen lectin using primer pair lect135f/lect135r (**A**, lanes 1–4), GMO allergen epsps using primer pair epsps114f/epsps114r (**B**, lanes 1–5) and epsps172f/epsps172r (**C**, lanes 1–5). Samples: (**A**) lane 1. soybean seeds flour, lanes 2–4. water (negative control); (**B**,**C**) Lanes 1–4. CRMs of Roundup Ready soybean (RRS) set: 0, 0.1%, 0.5%, 10%; lane 5. water (negative control); M. Molecular weight marker (Qiagen GelPilot 100 bp ladder).

The efficiency of newly designed primer pairs was tested separately for amplifying their targets by conventional uniplex/duplex PCRs (Figure 1 and Figure 2). Figure 1 shows an agarose gel electrophoresis of the PCR products of genes for endogenous soybean-specific allergens Gly m Bd 28K (Figure 1A) and Gly m Bd 30K (Figure 1B,C). The genomic DNA extracted from soybean flour was used as a template.

The single amplicon of the expected size was amplified in all soybean samples, in particular, primer pairs 28K86f/28K86r and 28K98f/28K98r targeted to the Gly m Bd 28K gene generated 86 bp and 98 bp fragments, respectively (Figure 1A, lanes 1–2, 4–5). However, the primer pairs 30K122f/30K122r, 30K129f/30K129r and30K118f/30K118r targeted to the Gly m Bd 30K gene produced 122 bp, 129 bp, and 118 bp amplicons, respectively (Figure 1B, 1C, lanes 1–2, 4–5). Moreover, a PCR band corresponding to the primer set 30K118f/30K118r exhibited higher intensity than all the other PCR products. This indicated the best sensitivity and efficiency of the PCR test with primers 30K118f and 30K118r in the assessment of soybean allergenicity. In addition, the absence of any amplification products in all negative controls (Figure 1, lanes 3, 6) indicated the absence of contaminating DNA.

Figure 2 shows agarose gel electrophoresis of PCR products of genes for soybean allergen lectin and GMO-specific potential allergenic protein epsps.

The primers lect135f and lect135r targeted to the lectin gene produced the 135 bp amplicon after amplification of the soybean genomic DNA, while the water control did not show any PCR product (Figure 2A, lanes 1–3). The amplifiability of the lectin-specific primer sets Lect101f/Lect101r producing 101 bp PCR product was investigated previously [33,38] and here these primers were used directly in multiplex PCR analysis.

Two newly-designed primer sets were examined for detection of exogenous GMO-specific *epsps* gene introduced in herbicide-tolerant GMOs such as RRS. Correspondingly, this approach can also be used for screening herbicide-tolerant GMOs. The expression of *epsps* gene gives potential allergenic protein 5-enolppyruvyl shikimate-phosphate synthase [23]. The epsps114f/epsps114r and epsps172f/epsps172r primer pairs directed to the *epsps* gene produced 114 bp and 172 bp PCR products, respectively (Figure 2B,C) in samples with genetically modified RRS DNA templates as expected. Moreover, the intensity of the PCR band increased correspondingly to the increased amount of transgenic material in the samples (Figure 2B,C lanes 2–4). In addition, the appearance of amplified product for 0.1% RRS template confirmed the suitability of these PCR tests for detection of herbicide-tolerant GMOs with high sensitivity (at least 0.1% GM material). Consistent with the high specificity of detection by this method, no PCR fragments were observed for samples with 0% RRS and water control (Figure 2, lanes 1, 5).

### 3.2. Development of Fourplex PCR

**Figure 3:**
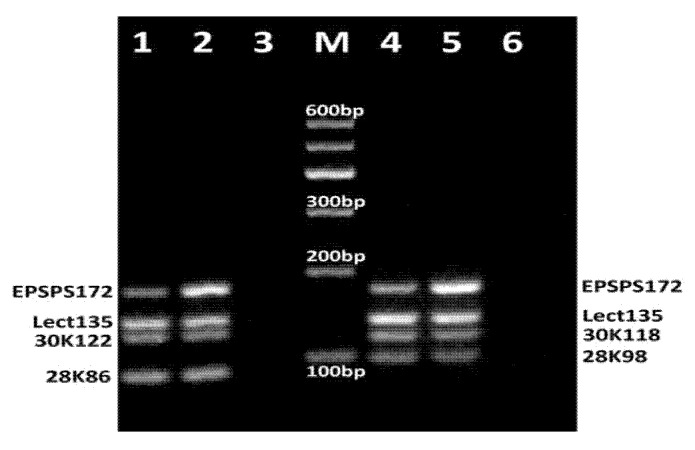
Fourplex PCRs for detection of GM Soybean allergens lectin, Gly m Bd 28K, Gly m Bd 30K and epsps using primer pairs lect135f/lect135r, 28K86f/28K86r, 30K122f/30K122r, epsps172f/epsps172r (N2) (lanes 1–3) and lect135f/lect135r, 28K98f/28K98r, 30K118f/30K118r, epsps172f/epsps172r (N1) (lanes 4–6). Samples: lanes 1, 4. CRM 1% RRS; lanes 2, 5. CRM 10% RRS, lanes 3, 6. water (negative control). M. Molecular weight marker (Qiagen GelPilot 100 bp ladder).

**Figure 4:**
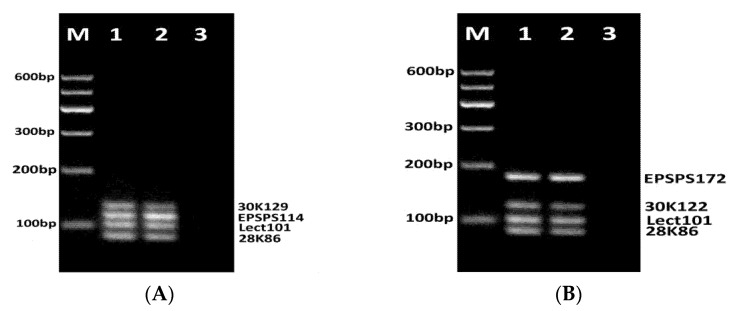
Fourplex PCRs for detection of GM Soybean allergens lectin, Gly m Bd 28K, Gly m Bd 30K and epsps using primer pairs 30K129f/30K129r, epsps114f/epsps114r, Lect101f/Lect101r, 28K86f/28K86r (N3) (**A**, lanes 1–3) and epsps172f/epsps172r, 30K122f/30K122r, Lect101f/Lect101r, 28K86f/28K86r (N4) (**B**, lanes 1–3). Samples: lane 1. CRM 1% RRS; lane 2. CRM 10% RRS, lane3. water (negative control). M. Molecular weight marker (Qiagen GelPilot 100 bp ladder).

Four fourplex PCR systems were designed with the following different combinations of primer pairs: Multiplex PCR (N1) with the primers: lect135f/lect135r, 28K98f/28K98r, 30K118f/30K118r, epsps172f/epsps172r; Multiplex PCR (N2) with the primers: lect135f/lect135r, 28K86f/28K86r, 30K122f/30K122r, epsps172f/epsps172r; Multiplex PCR (N3) with the primers: 30K129f/30K129r, epsps114f/epsps114r, Lect101f/Lect101r, 28K86f/28K86r; Multiplex PCR (N4) with the primers: epsps172f/epsps172r, 30K122f/30K122r, Lect101f/Lect101r, 28K86f/28K86r. After optimization of primer concentrations and PCR conditions, each of the fourplex PCR reactions produced four amplicons of expected sizes (Figure 3 and Figure 4).

Figure 3 shows agarose gel electrophoresis of PCR products for two fourplex PCRs (N1 and N2). The fourplex PCR (N2) of RRS DNA with primer sets epsps172f/epsps172r, lect135f/lect135r, 30K122f/30K122r and 28K86f/28K86r generated four expected amplicons such as 172 bp fragment of *epsps* gene, 135 bp fragment of lectin gene, 122 bp fragment of Gly m Bd 30K gene and 86 bp fragment of Gly m Bd 28K gene (Figure 3, lanes 1–2). However, the PCR bands revealed different intensities, namely 30K122 amplicon showed the least intensity. The fourplex PCR (N1) of RRS DNA using primer sets epsps172f/epsps172fr, lect135f/lect135r, 30K118f/30K118r and 28K98f/28K98r produced four expected amplified products such as 172 bp fragment of *epsps* gene, 135 bp fragment of lectin gene, 118 bp fragment of Gly m Bd 30K gene and 98 bp fragment of Gly m Bd 28K gene, whereby amplicon lect135 exhibited the highest intensity (Figure 3, lanes 4–5).

Four expected amplicons such as the 129 bp fragment of Gly m Bd 30K gene, the 114 bp fragment of *epsps* gene, the 101 bp fragment of the lectin gene, and the 86 bp fragment of Gly m Bd 28K gene were amplified by fourplex PCR of RRS DNA using primer sets 30K129f/30K129r, epsps114f/epsps114r, Lect101f/Lect101r, 28K86f/28K86r (Figure 4A, lanes 1–3). Moreover, the PCR amplicon bands exhibited almost similar yields for 1% RRS suggesting that this PCR test can detect all four allergens with similar efficiency. Four expected amplicons, such as the 172 bp fragment of *epsps* gene, the 122 bp fragment of Gly m Bd 30K gene, the 101 bp fragment of the lectin gene, and the 86 bp fragment of Gly m Bd 28K gene were amplified by fourplex PCR of RRS DNA with primer sets epsps172f/epsps172r, 30K122f/30K122r, Lect101f/Lect101r and 28K86f/28K86r. However, the 30K122 amplicon revealed the least intensity while the epsps172 PCR product showed the highest yield (Figure 4B, lanes 1–2). These data confirm previous results about the suitability of Lect101f and Lect101r primers for specific detection of soybean lectin gene [37,38].

In addition, the comparison of PCR products of 1% RRS and 10% RRS DNA templates in all fourplex PCRs (N1-N4) exhibited almost similar yield for soybean specific allergens (lectin, Gly m Bd 28K and Gly m Bd 30K), whereas epsps amplicons of 10% RRS DNA showed higher yield than the same amplicons of 1% RRS template (Figure 3 and Figure 4, lanes 1–2, 4–5). No amplification products were observed in negative control (water) samples (Figure 3 and Figure 4, lanes 3, 6).

### 3.3. Development of DNA Chips for GM Soya Allergens

**Figure 5:**
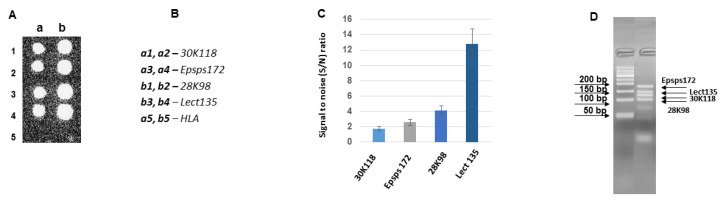
Hybridization of asymmetric multiplex PCR (N1) products with the probes Lect135, epsps172, 28K98 and 30K118 on a biochip. (**A**) Hybridization image (**a**,**b**) designations of the columns for the probe arrangement; (**B**) The arrangement of the probes on the biochips; (**C**) Analysis of hybridization; the hybridization signal intensities estimated as signal to noise (S/N) ratio. (**D**) Agarose gel electrophoresis of asymmetric PCR products. Total DNA per hybridization is 1300 ng, the amount of fluorescent dye Cy3 is 18 pmol.

In this study, four optimized multiplex PCRs were performed as asymmetric PCRs and analyzed for the development of a biochip coupled PCR approach. Figure 5 represents the result of the hybridization of asymmetric multiplex PCR (N1) products with the probes Lect135, Epsps172, 28K98, and 30K118. The human leukocyte antigen (HLA) gene fragment was a nonspecific negative control probe everywhere. According to Figure 5, the hybridization efficiencies of the probes Epsps172 and 30K118 were low. As the amplification efficiencies of the amplicons were identical, we suggest that it is an intrinsic property of the probes and/or a hybridizing target’s microenvironment.

**Figure 6:**
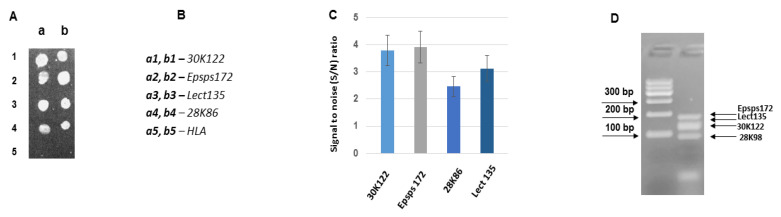
Hybridization of asymmetric multiplex PCR (N2) products with the probes Lect135, epsps172, 28K86 and 30K122 on a biochip. (**A**) Hybridization image (**a**,**b**) designations of the columns for the probe arrangement; (**B**) The arrangement of the probes on the biochips; (**C**) Analysis of hybridization; the hybridization signal intensities estimated as signal to noise (S/N) ratio. (**D**) Agarose gel electrophoresis of asymmetric PCR products. Total DNA per hybridization is 858 ng, the amount of fluorescent dye Cy3 is 12 pmol.

Figure 6 represents the result of the hybridization of asymmetric multiplex PCR (N2) products with the probes Lect135, Epsps172, 28K86, and 30K122. The composition of the N2 multiplex differs from the N1 multiplex by two components. The components Lect135 and Epsps172 are the same, but instead of 28K98, there is 28K86, while instead of 30K118 there is 30K122. The amplicons 28K86 and 28K98, as well as the amplicons 30K118 and 30K122, are from the different parts of the corresponding genes, and these gene segments are not overlapping. According to Figure 6, all probes show very effective hybridization capacity, and the probe Lect135 does not reveal a distinctly high hybridization efficiency as it was in N1; its hybridization efficiency is comparable with other probes in the N2 multiplex. As to the probe Epsps172, it is characterized by available hybridization efficiency in the N2 asymmetric multiplex product hybridization.

**Figure 7:**
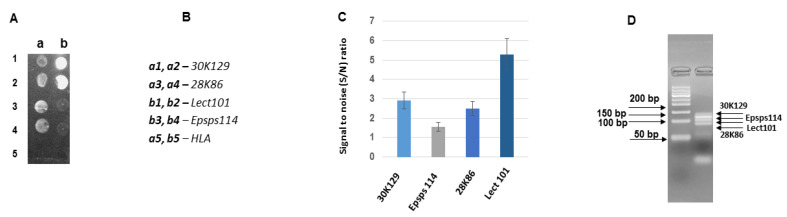
Hybridization of asymmetric multiplex PCR (N3) products with the probes Lect101, epsps114, 28K86 and 30K129 on a biochip. (**A**) Hybridization image (**a**,**b**) designations of the columns for the probe arrangement; (**B**) The arrangement of the probes on the biochips; (**C**) Analysis of hybridization; the hybridization signal intensities estimated as signal to noise (S/N) ratio. (**D**) Agarose gel electrophoresis of asymmetric PCR products. Total DNA per hybridization is 477 ng, the amount of fluorescent dye Cy3 is 6 pmol.

Figure 7 represents the result of the hybridization of asymmetric multiplex PCR (N3) products with the probes Lect101, Epsps114, 28K86, and 30K129. There is only one probe 28K86 in the composition N3, which was analyzed before in the composition N1. The amplicons Lect101 and Lect135, as well as the amplicons 30K118, 30K122, and 30K129, are from the different parts of the corresponding genes, and these gene segments are not overlapping. Concerning the amplicons Epsps114 and Epsps172, they are amplified from the same part of the gene and are overlapping. According to Figure 7, the hybridization efficiency of the probe Epsps114 was very low. We suggest that it is an intrinsic property of the probe and/or a hybridizing target’s microenvironment because the amplification efficiency of this amplicon was very high.

**Figure 8:**
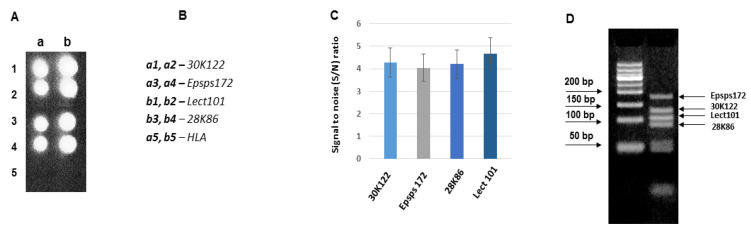
Hybridization of asymmetric multiplex PCR (N4) products with the probes Lect101, epsps172, 28K86 and 30K122 on a biochip. (**A**) Hybridization image (**a**,**b**) designations of the columns for the probe arrangement; (**B**) The arrangement of the probes on the biochips; (**C**) Analysis of hybridization; the hybridization signal intensities estimated as signal to noise (S/N) ratio. (**D**) Agarose gel electrophoresis of asymmetric PCR products. Total DNA per hybridization is 1340 ng, the amount of fluorescent dye Cy3 is 18 pmol.

Figure 8 represents the result of the hybridization of asymmetric multiplex PCR (N4) product with the probes Lect101, Epsps172, 28K86, and 30K122. The multiplex PCR (N4) consists of the amplicons that have revealed similar hybridization efficiency with the corresponding probes in the above-mentioned multiplex PCRs. Therefore, a comparable hybridization efficiency of all probes was expected for N4 products. Indeed, as shown in Figure 8, all probes show very effective and similar hybridization capacity.

### 3.4. Analysis of the Developed DNA Chips

**Figure 9:**
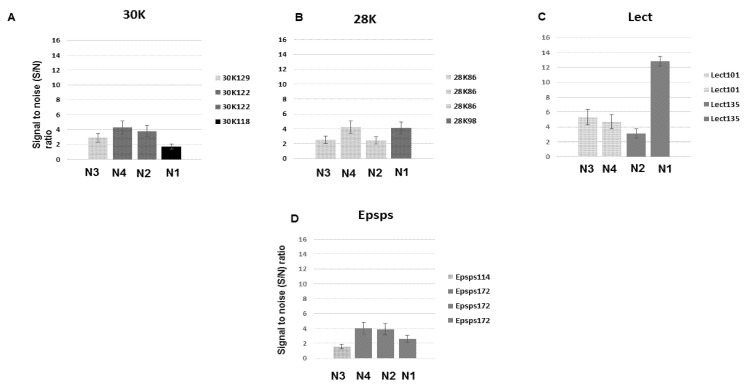
The analysis of DNA chips for GM soya allergens 30K (**A**), 28K (**B**), lectin (**C**) and epsps (**D**).

The analysis of DNA chips for GM soya allergens (30K, 28K, Lectin and epsps) is presented in Figure 9. The probes 30K, were designed for the detection of the soya allergen gene-Glycine max Gly m Bd 30K allergen (P34) gene. The PCR products 30K118, 30K122 and 30K129 are amplified from different parts of the gene: the amplicon 30K118 spans the region between the base-pairs 304–421, the amplicon 30K122 spans the region between the base-pairs 1108–1229, and the amplicon 30K129 spans the region between the base-pairs 106–234. The hybridization capacity of the probe 30K122 is closely related in both multiplex combinations N2 and N4, and differs from the hybridization capacity of the probes 30K118 (N1), and 30K129 (N3) (Figure 9A).

The hybridization capacity of the probes 28K86 and 28K98, designed for the detection of the soya allergen gene-Glycine max cultivar GX Gly m Bd 28K allergen gene, is presented in Figure 9B. The PCR products 28K86 and 28K98 are amplified from different parts of the gene: the amplicon 28K86 spans the region between the base-pairs 1995–2080; the amplicon 28K98 spans the region between the base-pairs 1830–1927. The hybridization capacity of the probe 28K86 is similar in the multiplex combinations N2 and N3 and is almost twice higher in the multiplex combination N4. The hybridization efficiency of probe 28K98 (N1) is closely related to probe 28K86 in N4 composition.

The hybridization capacity of the probes Lect101 and Lect135, designed for the detection of the soya allergen gene-Soybean lectin (Le1) gene, is presented in Figure 9C. The PCR products Lect101 and Lect135 are amplified from different parts of the gene: the amplicon Lect101 spans the region between the base-pairs 1255–1274, the amplicon Lect135 spans the region between the base-pairs 1611–1745. As the result, the hybridization efficiency of the PCR product Lect101 is similar in two studied multiplex combinations (N3 and N4), and different from the hybridization efficiency of the PCR product Lect135 (N1 and N2).

The probes Epsps114 and Epsps172 are designed for the detection of the GM soya specific gene-Glycine max transgenic cp4epsps gene for 5-enol-pyruvylshikimate-3-phospate synthase class 2 precursor (Figure 5D). The PCR products Epsps114 and Epsps172 are amplified from the same part of the gene: the amplicon Epsps114 spans the region between the base-pairs 850–963, the amplicon Epsps172 spans the region between the base-pairs 791–962. The amplification products are overlapping; however, they are hybridized with the different probes, and their target-labeled strains are opposite. According to Figure 5D, the hybridization capacity of the probe Epsps172 is closely related in the three multiplex combinations (N1, N2, N4). The hybridization effectiveness of the probe Epsps114 (N3) is very low and below the threshold of detection. The threshold of detection is S/N > 1.5.

## 4. Discussion

The development of effective methods for assessment of both the endogenous and exogenous potential allergens of genetically modified soybean are in urgent need to support the regulatory requirements and to maintain food safety and public health [7,8,23,26]. The most widely distributed GM event Roundup-ready soybean was chosen as an object of this study. We investigated three important soybean allergens as endogenous allergens based on the available gene sequences. These allergens are 26kDa glycoprotein Gly m Bd 28K, peptidase C1 Gly m Bd 30K, and lectin. The proteins Gly m Bd 30K and Gly m Bd 28K are recognized as major soybean allergens. The Gly m Bd 30K protein (vacuolar storage protein P34) is a unique member of the papain family of cysteine proteases. Gly m Bd 28K/P28 is a vicilin-like glycoprotein with a molecular weight of 26,000 [13]. Lectins or carbohydrate-binding proteins are widely distributed in seeds and vegetative parts of edible plant species. They are considered as potential food allergens, however, more investigations are needed to fully assess their allergenicity [40]. Soybean lectin is a species-specific single-copy gene, which is usually used as a target for the allergenic soybean detection [21]. RRS belongs to the herbicide-tolerant GM crops because a 5-enolppyruvyl shikimate-phosphate synthase (*epsps*) gene from soil bacteria *Agrobacterium tumefaciens* was introduced into soybean genome. Consequently, RRS may contain potential allergenic epsps protein due to the expression of the newly introduced gene. Thus, epsps protein was studied as potential exogenous allergen of RRS.

To devise a fast and reliable method for allergen detection we combined multiplex PCR with DNA chip technology. This approach is considered innovative, sensitive, selective, low cost, and fast [27,30]. The procedure included several main stages, such as bioinformatics design of allergen-specific PCR primers and probes; DNA purification and amplification by uniplex and multiplex PCR; verification of PCR products by agarose gel electrophoresis; development and hybridization of the DNA chips. The PCR methods and DNA chips were optimized using the RRS standards (CRMs). The obtained results have revealed that eight newly designed PCR primers and optimized PCR protocols applied in this study are useful for reliable detection of genes for three soybean allergen such as Gly m Bd 28K, Gly m Bd 30K, and lectin as well as GMO-specific potential allergenic *epsps* gene. Consequently, they were applied in multiplex PCR analysis. The detected allergen-specific PCR fragments can be considered as novel DNA markers as they are distinguished from the allergen DNA markers described previously for soybean and GMO [9,19,20,21]. These DNA markers are 86 bp and 98 bp amplicons for Gly m Bd 28K; 129 bp, 118 bp, and 122 bp amplicons for Gly m Bd 30K; 135 bp amplicon for lectin as well as 114 bp and 172 bp amplicons for *epsps* gene.

In this study, fourplex PCRs were designed and optimized for simultaneous detection of three soybean allergens such as Gly m Bd 28K, Gly m Bd 30K, and lectin together with GMO-specific epsps protein using the primer pairs described in Table 1. The outcomes of the study indicate that the developed fourplex PCR methods enable us to detect GM soybean allergens with a high specificity, whereby the fourplex PCR using the primer sets 30K129f/30K129r, epsps114f/epsps114r, Lect101 f/Lect101r, 28K86f/28K86r (N3) shows the highest reliability.

DNA biochips based on DNA–DNA hybridization and DNA amplification have become the leading diagnostic technologies. These technologies can be used separately or in combinations [41,42]. Direct hybridization of PCR amplification products is not optimal, because re-annealing of double-stranded (ds) PCR products can compete with hybridization of the target strand to the probe immobilized on the matrix, reducing or even abolishing signal intensity. For this purpose, we have developed and optimized an asymmetric multiplex PCR, where forward and reverse primers are in non-equal quantity, and as a result fluorophore-labeled single-stranded (ss) PCR products for the detection of GM soybean allergens are obtained. Notably, the asymmetric multiplex PCR coupled with a DNA biochip approach has been used previously for the discrimination of *Mycobacterium tuberculosis* and non-tuberculous mycobacterial strains [43].

Pseudo-3D structures (dendrimers) were manufactured as solid matrixes for a biochip and used in this study. Dendrimers represent repetitively branched polymer structures with numerous activated terminal functional groups [44,45]. A 3D format considerably increases the available surface area and allows the deposition of higher probe quantities, providing for a higher sensitivity of the biochip analysis. As a result, a fairly low concentration of fluorescent dye Cy3 such as 6 pmol is detectable on a biochip. The proposed multiplex PCR coupled with a low-density biochip approach provides an opportunity for the semi-quantitative screening of up to 100 features of both the GMO and the allergens simultaneously in food products, seeds, etc. In addition, highly effective and cost-saving in-house manufactured 3D dendrimeric biochip matrixes are provided. Therefore, the developed technical approach allows us to reduce the time and price of allergen analysis.

One of the advantages of multiplex PCR coupled with a biochip is that it does not depend on the size of the amplification products for product identification. A relevant example is the multiplex N2 coupled with a biochip. The amplicons 30K122 and Lect135 are not distinguishable on agarose gel, however, they are discriminated by subsequent hybridization with the probes. The increasing set of primer pairs in multiplex PCR greatly affects the efficiency of PCR, but this problem can be overcome by the analysis of a combination of several multiplex PCRs on one biochip. High detection throughput of amplicons with the close or even identical size is the other advantage.

The hybridization efficiency of amplicons depends on the hybridization capacity of the probe and hybridizing target’s microenvironment. The observed variation of hybridization signal intensity between the spots/probes in multiplex PCRs N1, N2, and N3, could be attributed to the different hybridization capacities of the probes.

It is noteworthy, that hybridization capacity is a property that depends on many factors, including the secondary and tertiary structure of the target DNA and the probe; the position of the short capture probe on the target nucleic acid, etc. [46]. In spite of the adjustment of characteristics of the probes such as melting temperature, G+C content, and developing new algorithms considering the probe-surface distance, external electrostatic fields, and probe-surface density [47], the hybridization behavior of small oligonucleotides arrayed on glass slides is currently difficult to predict.

The amplicon 30K122 is present in two multiplex PCRs (N2 and N4), and its hybridization efficiency with the subsequent probe in both multiplex PCRs is the same. The same concerns the amplicons Lect101 and Epsps172. The amplicon Lect101 is present in two multiplexes PCRs (N3 and N4), and reveals similar hybridization efficiency. The amplicon Epsps172 is present in three multiplex PCRs (N1, N2, and N4), and its hybridization efficiencies in all cases are closely related. Regarding the amplicons 28K86 and Lect135, their hybridization efficiency differs in different multiplex compositions. The amplicon 28K86 hybridizes with the same efficiency in multiplex PCRs N3 and N2, and with a different efficiency in multiplex N4. The amplicon Lect135 is characterized by significantly different hybridization efficiency in the multiplex PCRs N2 and N1. We propose that the different hybridization efficiency of the same amplicon in the different multiplex compositions could be the result of microenvironment influence, as the hybridization capacity of the probe should be the same.

The amplicon Epsps114 demonstrated very low hybridization efficiency. The amplicons Epsps172 and Epsps114 are derived from the same part of the gene and are overlapping; however, they are hybridized with different probes, and their target strands are opposite. Therefore, the optimization of the approach involves the indication of probes unsuitable for inclusion in the biochip.

The optimization of multiplex PCR coupled with a biochip includes the empirical selection of multiplex PCR composition, revealing the best hybridization profile with the probes characterized by the identical hybridization capacity (N4). This provides an opportunity for semi-quantitative screening of the allergens features in food products, namely, the validation of the prevailed or absent GM modifications and allergens.

## 5. Conclusions

In this paper, a fourplex asymmetric PCR coupled with a DNA biochip approach was successfully developed for the detection of endogenous and exogenous allergens in GM soybean. For this purpose, transgenic herbicide-resistant soybean event Roundup Ready soya carrying the 5-enolppyruvyl shikimate-phosphate synthase (*epsps*) gene from *Agrobacterium tumefaciens* was investigated. The set of certified reference materials consisting of 0, 0.1%, 0.5%, and 10% RRS was used for the development and optimization of PCR systems and DNA chips. The uniplex and multiplex PCR systems as well as DNA chips using new primer pairs and probes targeting major soybean allergens, such as Gly m Bd 28K, Gly m Bd 30K and lectin as well as *epsps* gene were developed and optimized. The asymetric multiplex PCR products were very effective in hybridization with suitable labeled biochip probes. The results obtained demonstrated high specificity and sensitivity of the new approach, enabling simultaneous detection of three major soybean allergens, namely Gly m Bd 28K, Gly m Bd 30K, and lectin as well as potential allergenic *epsps* protein expressed in herbicide-tolerant roundup ready GMOs. The methods obtained can be used for reliable, fast, and cheap testing of allergens in food products.

## Figures and Tables

**Table 1:** PCR primers and DNA chip probes used in this study.

Primer Probe	Sequence 5′→3	5′- Label	Target Gene	Amplicon Size (bp)	Reference
28K86f 28K86r 28K86 probe	ACTGCATGGAGGCGAGTATC GGACATGTTTGCATACCGCT CCACTCAGCGAACCGGATATTGGA	CY3 AC6	Gly m Bd 28K	86	This study
28K98f 28K98r 28K98 probe	GGAGGAGAAGCAAACAAGTAGG GGGAACCAGCAGTGTCTTT ATG GAG GAA GCT CTT GGA AAC CGT	CY3 AC6	Gly m Bd 28K	98	This study
30K129f 30K129r 30K129 probe	GCTACGAGGGAACTTCTTCAGT AGGAAACCCATAACTTGGTGGA ACGACATGCTACAAGTGAAGTGACCA	CY3 AC6	Gly m Bd 30K	129	This study
30K118f 30K118r 30K118 probe	GGACCTTGACCTAACCAAGTT CTCAAGTCTCTTTGCCTCTTCT ATGGAAGAGTGAGCATGGACGTGT	CY3 AC6	Gly m Bd 30K	118	This study
30K122f 30K122r 30K122 probe	CAGGAGACCTTGTTAGCCTTT GGCAATCCCACCATGTTCTA AGACTGTGTGGAAGAAAGCGAAGGT	CY3 AC6	Gly m Bd 30K	122	This study
Lect101f Lect101r Lect101 probe	ACGGCACCCCAAAACCCTCG GGAAGCGGCGAAGCTGGCAA CCGGTAGCGTTGCCAGCTTC	CY3 AC6	lectin	101	[38]
lect135f lect135r lect135 probe	TCACAGAGAACCAGCAATATCC AGACCAAGAAAGCACGTCAT AGACCAAGAAAGCACGTCAT	CY3 AC6	lectin	135	This study
epsps114f epsps114r epsps114 probe	TACGATTTCGACAGCACCTTC GTCACCGTCTTCCGATTTCA TTGAACCCGCTGCGCGAAATG	CY3 AC6	epsps	114	This study
epsps172f epsps172r epsps172 probe	CGCTCGATTTCGGCAATG TCACCGTCTTCCGATTTCAC AAGGTGCTGTCGAAATCGTAGACCC	CY3 AC6	epsps	172	This study

## Data Availability

All data generated and analyzed during this study are included in the present article.
